# Eating to Feel Better: The Role of Comfort Eating in Chronic Pain

**DOI:** 10.1007/s10880-025-10064-6

**Published:** 2025-02-22

**Authors:** Claudia Roche, Amy Burton, Toby Newton-John

**Affiliations:** University of Technology Sydney, Sydney, Australia

**Keywords:** Chronic pain, Comfort eating, Pain-induced comfort eating, Overeating, Obesity

## Abstract

Research has identified that individuals with chronic pain comfort eat in response to their pain, however, little is known about the function that comfort eating serves for chronic pain patients. Given the synergistic relationship between higher body weight and chronic pain, it is important to further understand the role and impact of eating behaviours, such as comfort eating, for individuals with chronic pain. This study aimed to investigate the perceived function of pain-induced comfort eating for chronic pain sufferers. Adult participants (*N* = 141) with chronic pain were recruited through online advertisements. Participants completed self-report questions on an online survey platform. Over two-thirds of the sample identified with engaging in comfort eating in response to chronic pain flare-ups. Results revealed that the most endorsed function of pain-induced comfort eating was ‘to have a pleasant experience’ (51.8%), followed by ‘distraction’ (49.6%) and ‘to reduce emotions’ (39%). This study provides further evidence that comfort eating is common amongst individuals with chronic pain and sheds light on the perceived function of comfort eating for those who are managing chronic pain. Given the potential impact on outcomes for chronic pain patients, future studies should further investigate the relationship between comfort eating and chronic pain.

Chronic pain, defined as pain that persists for at least three months (Cohen et al., 2021), is a significant public health problem affecting upwards of 20% of the population globally (Zimmer et al., 2022). This equates to one in five individuals living with persistent pain, and this number is expected to grow (CDC, 2020; Yong et al., 2022). Obesity, defined as a Body Mass Index (BMI) of 30 or above, is also prevalent with recent data indicating that over 40% of American adults have obesity (Emmerich et al., 2024) and over half of the adult population worldwide are classified as having a body weight that falls into the overweight or obese ranges (i.e. a BMI of 25 or above; OECD, 2024). Both obesity and chronic pain are associated with significant individual and public health concerns (Okifuji & Hare, 2015). Obesity commonly co-occurs with a range of physical and psychiatric co-morbid conditions (Bae et al., 2024; Fulton et al., 2022; Guh et al., 2009; Ortega et al., 2016), while chronic pain is associated with impaired physical and social functioning as well as mood and anxiety disorders (Turk et al., 2016).

It is therefore not surprising that chronic pain and obesity have been described as “two colliding epidemics” (Allen et al., 2016) with the prevalence of pain in individuals with obesity as high as 40% (Mills et al., 2019). The synergistic nature of chronic pain and obesity is particularly concerning as each condition is thought to intensify the negative health outcomes of the other (Chin et al., 2020). Moreover, individuals with obesity experience higher levels of pain intensity and interference (Basem et al., 2021) and are less responsive to chronic pain treatments (Ewald et al., 2016). Therefore, understanding factors that may influence this relationship is imperative. Pain-induced comfort eating (PICE), defined as the overconsumption of palatable and typically energy-dense foods for the specific purpose of ameliorating the discomfort associated with the experience of pain (O’Loughlin & Newton-John, 2019), has been identified as one such factor.

## Pain-Induced Comfort Eating

Comfort eating is eating for the purpose of soothing feelings of upset or discomfort in the absence of hunger (O’Loughlin & Newton-John, 2019). Comfort eating has been found to be a common coping strategy for those living with chronic pain amongst both qualitative and quantitative studies (Chisholm et al., 2016; Choi et al., 2014; Godfrey et al., 2018; Janke & Kozak, 2012; Janke et al., 2016; Masheb et al., 2020; O'Loughlin & Newton-John, 2019). However, *why* individuals engage in comfort eating when experiencing pain flare-ups is not yet well-understood. Pain is a powerful motivator which demands attention such that individuals are driven to escape tissue-damaging stimuli or minimise pain sensations experienced (Eccleston & Crombez, 1999; Phelps et al., 2021). As such, individuals seek a variety of ways to avoid or reduce its aversiveness (Ersek et al., 2006). Comfort eating has been conceptualised as one such form of experiential avoidance in which individuals may temporarily attempt to avoid unpleasant internal experiences, such as negative emotions or physical sensations, regardless of the long-term consequences (Litwin et al., 2017).

The use of food as means to escape from uncomfortable emotional experiences has also been well-documented within the binge eating literature, as noted in the Escape Model of Binge Eating (Heatherton & Baumeister, 1991). It could be hypothesised that food consumption is a strategy by which individuals attempt to escape or avoid the pain in the short term. Participants in Janke and Kozak's (2012) study suggested that comfort eating served as a distraction from their pain; however, O’Loughlin and Newton-John (2019) did not find a measure of experiential avoidance to be a significant mediator of the chronic pain and PICE relationship. This discrepancy in findings may be due to the way in which experiential avoidance and distraction are defined within the studies. Qualitatively, distraction may both serve to cope with unwanted internal experiences as well as alter the pain experience (Burton & Abbott, 2017; Foo & Mason, 2009; Heatherton & Baumeister, 1991; Kakeda et al., 2010), hence more research is needed to better understand the function of comfort eating for those with chronic pain.

Engaging in comfort eating as an avoidance strategy may be further reinforced by the analgesic effects of food. Some promising findings from animal and human studies have indicated that consuming food high in fat, sugar or salt produces analgesic effects (Foo & Mason, 2009; Kakeda et al., 2010). More specifically, consuming such comfort foods reduces pain symptoms and enhances pain tolerance (Anjana & Reetu, 2014; de Freitas et al., 2012; Foo & Mason, 2009). Food consumption has been shown to influence internal experiences including improving negative affect in the short-term (Gibson, 2006; Macht & Mueller, 2007) as well as psychological stress (Markus et al., 2000; Wallis & Hetherington, 2009). This aligns with theoretical models of binge eating in which stress or negative affect triggers food consumption as a maladaptive coping mechanism for affect regulation (Burton & Abbott, 2019; Cooper et al., 2004; Fairburn et al., 2003; McManus & Waller, 1995; Stice et al., 1998; Williamson et al., 2004). In their study, O’Loughlin and Newton-John (2019) found that stress was a significant mediator in the chronic pain and comfort eating relationship. Based on the above research, it seems reasonable that, similar to binge eating, PICE could play a role in modulating both the unpleasant sensory input and the emotional distress associated with experiencing a pain flare-up, however, further research is needed to explore the function of this potentially maladaptive coping strategy.

It is important to note that while chronic pain has been identified as a common co-morbidity of binge eating disorder (Olguin et al., 2017), the relationship between binge eating and chronic pain has not yet been explicitly investigated. To date, the most relevant study exploring these constructs is a qualitative study of 30 individuals with BMIs greater than 25 experiencing non-cancer pain (Janke & Kozak, 2012). In this study, participants described responding to pain through consuming larger quantities of high sugar, high fat food with many participants using the term “binge” to describe their eating-related behaviour. The authors noted that many responses reflected characteristics of binge eating episodes as defined in the DSM-5, however, this was not investigated specifically (Janke & Kozak, 2012). Additionally, the generalisability of Janke and Kozak’s (2012) study is limited due to a small sample size and the exclusion of participants with a BMI of less than 25. Among quantitative studies, both Godfrey et al. (2018) and Janke et al. (2016) found that chronic pain intensity and presence of chronic pain were associated with increased levels of emotional eating. Further research is needed to understand the nature, role and function of food consumption habits for people experiencing chronic pain.

## The Present Study

This study therefore aimed to explore the role and function of PICE within a community chronic pain sample. Specifically, this research aimed to measure the frequency of PICE within a community-based chronic pain sample and to explore the perceptions of the function of PICE for people with chronic pain. Based on the previous research outlined above, we expected to observe a high rate of engagement in PICE behaviours (> 50% of the sample) and that participants would report that they engage in PICE as a way of coping with the physical pain and/or the emotional distress related to chronic pain flare-ups. Specifically, based on the literature, we expected that participants would most strongly endorse the use of PICE as a means of providing a distraction from the pain or to assist in reducing negative affect associated with the pain.

## Method

### Participants

Eligible participants included those over 18 years who reported experiencing chronic pain (non-malignant pain persisting for three months or longer) had been diagnosed with chronic pain by a healthcare professional and reported currently residing in Australia. There were no additional exclusion criteria related to individuals. Participants were recruited from online advertisements on social media and Australian chronic pain organisation websites.

The total sample consisted of 141 participants (*M*_*age*_ = 41.40 years, *SD* = 13.23 years, *range* = 20–78 years), of which 101 (71.6%) identified as female, 36 (25.5%) identified as male and 4 (2.8%) identified as non-binary or ‘other’ gender. Regarding ethnicity, 121 (85.8%) were Caucasian, 7 (5%) were Indigenous Australian, 4 (2.8%) were Asian, 4 (2.8%) were African and 5 (3.5%) identified as ‘other ethnicity’. The mean BMI of the sample was 28.98 kg/m^2^ (*SD* = 8.13 kg/m^2^, *range* = 14.88–56.97 kg/m^2^). It was found that 2 (1.4%) were in the underweight BMI range, 44 kg/m^2^ (31.2%) were in the normal BMI range, 53 kg/m^2^ (37.6%) were in the overweight BMI range and 42 kg/m^2^ (29.8%) were in the obese BMI range.

## Measures

### Qualifying Questions

Ineligible participants were screened out through questions that assessed age, location and chronic pain status.

### Demographics

Author-constructed items assessed the following: age, gender, employment, ethnicity, education, living arrangements, chronic pain details, medication use, weight (in kilograms) and height (in centimetres). BMI was calculated and interpreted using the Centers for Disease Control and Prevention (CDC) guidelines; weight in kilograms (kg) divided by height in metres (m) squared, where a BMI less than 18.5 falls in the underweight range, a BMI of 18.5 to 24.9 is considered in the normal range, a BMI of 25 to 29.9 is considered to be in the overweight range and a BMI of over 30 is considered to be in the obese range (CDC, 2024).

### PICE

PICE was assessed using the Pain-Induced Comfort Eating Scale (PICES; Burton et al., 2023). This self-report questionnaire contains four items with participants asked to select a response based on the last three months. In item 1, participants are asked to respond to “How often do you use food as a way of coping with flare-ups of your chronic pain?” using an 8-point Likert scale, ranging from 0 (*never*) to 8 (*multiple times a day*)*.* Item 2 (“When experiencing pain flare ups, I eat more of my favourite foods to make myself feel better”) and Item 3 (“When experiencing pain flare ups, I eat more than I usually do”) ask participants to rate responses on a 4-point Likert scale, from 1(*not at all*) to 4 (*a lot*)*.* Item 4 asks participants to list the types of foods consumed when eating to cope with pain. Scores on item 1 to 3 are summed, with a minimum score of 3 and maximum of 16. Higher scores on the PICES indicate higher severity of PICE. The PICES has been shown to have good internal consistency and moderate to strong construct validity within a community chronic pain sample (Burton et al., 2023) and demonstrated good reliability within this study ($\alpha =.83)$.

### Function of PICE

Two author-constructed items were used to understand the reason why participants engaged in comfort eating. Participants were asked to tick all that apply in response to the question, *when I am in pain, I eat in order to*, in which, nine options were provided (e.g. *reduce my pain*). A blank text box was also provided for participants to note any reasons not identified in the previous question.

## Design and Procedure

The study utilised a cross-sectional online survey design and the study was approved by the UTS Human Research Ethics Committee (ETH22-7375). Online advertisements in the form of a flyer were posted to relevant social media sites (e.g. Australian-based Facebook groups and Reddit communities for people experiencing chronic pain) and also on relevant Australian chronic pain organisation websites (e.g. Chronic Pain Australia). After viewing the study flyer online, interested individuals followed a link to the study information (hosted on the Qualtrics survey platform). A participant information statement was then presented, and participants were required to indicate consent prior to proceeding. Qualifying questions screened out those not appropriate for the study (i.e. participants who did not have a diagnosis of chronic pain, participants not residing in Australia and participants not over the age of 18 years were exited from the survey). Eligible participants were invited to complete the survey-based study via the Qualtrics survey platform. Participants completed the online test-battery of measures and were provided with a debriefing statement on completion. At completion, participants were eligible to receive either a $15 e-gift card or to enter a draw for a chance to receive one of three $50 e-gift cards in recognition of their time.

## Analysis

Data analysis was conducted using SPSS Version 27. A data analysis plan to test the pre-determined study hypotheses was specified prior to the commencement of data collection. Missing data were handled using listwise deletion; complete data for the whole questionnaire were retained, any incomplete responses were removed prior to analysis. Out of the total *N* = 141 who completed the demographic items and the function of PICE questions, *n* = 134 completed all four items of the PICES (5% incomplete/missing data). Descriptive analyses were computed to characterise the sample. Frequency statistics were obtained to determine the most rated functions that comfort eating served for participants.

## Results

### Socio-Pain Demographics

Table 1 lists demographic characteristics and chronic pain information of the sample. 17% of the sample reported being unemployed due to pain with 61.7% reporting living with a spouse or partner. The mean duration of chronic pain was 7.8 years.

**Table 1:** Socio-pain demographic characteristics of the sample

	*n*	*%*	*M*	*SD*	*Range*
Highest level of education completed					
Post-graduate degree	50	35.5			
Bachelor’s degree	45	31.9			
Completed secondary schooling	33	23.4			
Between 9 and 11 years of education	13	9.2			
Living Arrangements					
Living with spouse/partner	87	61.7			
Living alone	22	15.6			
Living with family members/friends	32	22.7			
Employment status					
Full-time work	52	36.9			
Part-time work	25	24.8			
Home duties	4	2.8			
Unemployed due to pain	24	17			
Retired	7	5			
Other	19	13.5			
Duration of chronic pain (in months)			93.35	101.60	4–515
Number of pain sites			3.8	1.72	1–6
Main pain site					
Head/face	6	4.3			
Upper limb/hand	13	9.2			
Lower limb/foot	17	12.1			
Abdomen/pelvis	32	22.7			
Upper back/neck	18	12.8			
Lower back	32	22.7			
Other	23	16.3			
Previous surgery to treat chronic pain					
Yes	55	39			
No	86	61			
Medication Usage					
None	17	12.1			
Over-the-counter Medications	20	14.2			
Prescription Medications	104	73.8			
Medication Type					
Opioids	73	51.8			
Non-steroidal anti-inflammatories	76	53.9			
Benzodiazepines	42	29.8			
Anti-depressants	50	35.5			
Anti-convulsants	36	25.5			
Other	40	28.4			
Self-reported pain relief provided by medication in the last week			55.76	25.27	0–100
Psychological engagement to support pain management					
Currently engaged	47	33.3			
Engaged in past	57	40.4			
Never engaged	37	26.2			

*N* = 141 except for self-reported pain relief from medication, which was only displayed to participants who reported taking medication (*n* = 118)

### PICE

A total of 134 participant completed the PICES and a mean score of 9.5 (standard deviation of 4.3) was observed in the sample. Results indicated that over 70% of the sample reported using food as a way of coping with pain at least once a month (70.1%). Additionally, 64.2% reported engaging in PICE at least once a fortnight (period of 14 days). More than two-thirds of the sample reported eating more than they usually do when experiencing pain flare-ups (69.4%), and over three-quarters of the sample reported they eat more of their favourite foods to make themselves feel better when experiencing pain flare-ups (82.1%). Table 2 shows the frequency of self-reported PICE.

**Table 2:** Frequency of Pain-induced comfort eating

	*n*	*%*
Never	26	18.4
Less than once per month	14	9.9
Once a month	8	5.7
Once a fortnight	9	6.4
Once a week	20	14.2
Several times a week	28	19.9
Once a day	9	6.4
Multiple times a day	20	14.2

*n* = 134

### Function of PICE

As shown in Table 3, the most reported reasons participants identified for engaging in PICE included eating to provide a pleasant experience (51.8%), as a form of distraction (49.6%) and as a way in which to reduce emotions (39%).

**Table 3:** Responses to ‘When I am in pain, I eat in order to…’

	*n*	*%*
Give myself a pleasant experience	73	51.8
Distract myself	70	49.6
Reduce my emotions	55	39.0
To do something I can control	40	28.4
Forget my pain is there	39	27.7
Reduce my pain	34	24.1
None of these, I tend to eat less when in pain	26	18.4
Numb out	22	15.6
None of these, I tend to eat as usual when in pain	16	11.3

*N* = 141

Overall, participants who did endorse engaging in PICE reported a mean of 2.0 (SD = 1.8, Mode = 1.0) functions. Thirty-six participants endorsed only one function of PICE from the list, seventeen participants endorsed two functions, twenty-five participants endorsed three functions, fifteen participants endorsed four functions, nine participants endorsed five functions and six participants endorsed six functions. Zero participants endorsed all seven functions of PICE in the presented list.

## Discussion

Over two-thirds of participants endorsed comfort eating in response to chronic pain flare-ups with nearly two-thirds of the sample reporting engaging in PICE at least once every 2 weeks. These findings provide further evidence that comfort eating is a common and frequently used strategy to cope with chronic pain and is in line with the previous research suggesting that PICE is common among chronic pain samples (Chisholm et al., 2016; Choi et al., 2014; Godfrey et al., 2018; Janke & Kozak, 2012; Janke et al., 2016; Masheb et al., 2020; O’Loughlin & Newton-John, 2019). Interestingly, a minority (18.4%) of the sample reported eating less in response to pain. As the current study only focussed on comfort eating, it may be important for future research to ensure that all aspects of disordered eating, including under-eating, are explored.

Results exploring the function of PICE indicated that just under half of the sample reported they engage in PICE as a form of distraction, and more than one-third endorsed engaging in PICE to reduce their emotions. These functions are in line with the theoretical positions that suggest that comfort eating is a form of experiential avoidance (Litwin et al., 2017) and a way to regulate affect (Burton & Abbott, 2017; Heatherton & Baumeister, 1991; O'Loughlin & Newton-John, 2019). However, the most endorsed function was comfort eating to provide a pleasant experience, which was endorsed by just over half the sample. Among qualitative research, participants with chronic pain have reported eating as an activity that brings regular and reliable pleasure (Janke & Kozak, 2012), however, this function has been largely overlooked in research to date. Of relevance, eating to provide a pleasant experience may be influenced by eating beliefs and eating-related expectancies commonly identified as maintaining factors in transdiagnostic binge eating (Burton & Abbott, 2019; De Young et al., 2014). More specifically, positive (e.g. “eating makes me feel better”) and permissive beliefs (e.g. “I deserve to have a pleasure like binge eating”), and the eating-related expectancy that “eating is pleasurable and useful as a reward” have been linked to binge eating episodes (Cooper et al., 2004; De Young et al., 2014) and appear to align with the pleasure function in this study. Future research should assess eating beliefs and eating-related expectancies which might serve to maintain comfort eating in chronic pain samples, particularly as cognitions are a modifiable factor that could be directly targeted within treatment. Additionally, it is important to consider the high rates of co-morbid depression in those with chronic pain (Rayner et al., 2016). Using food to provide pleasure may be one way in which individuals attempt to manage their mood (Janke & Kozak, 2012), however, more research is needed to understand how mood may influence PICE. Further, given that recent research on emotional eating has identified the importance of assessing under-eating and positive affect, it will be important that future research endeavours to understand how PICE, emotional eating, as well as dietary restriction in response to pain, influences experiences of both positive and negative affect (Barnhart et al., 2020; Dixit et al., 2023). While our results indicated that much of the sample endorsed engaging in comfort eating when experiencing pain flare-ups, it remains unclear whether physical pain itself is the trigger for PICE. We suspect that the relationship between chronic pain, mood and comfort eating is more complicated, and it is likely that comfort eating may serve multiple functions, with mood and physical pain both influencing coping behaviours. Future research should aim to disentangle physical pain and mood via such methods like ecological momentary studies that can distinctively measure these factors and the way in which they both relate to comfort eating following pain flare-ups.

## Limitations and Future Directions

Results of this study need to be interpreted in the context of its limitations. Although this study used a relatively large sample size, the sample population included an online sample consisting of largely Caucasian, female participants, thus generalisation to all individuals with chronic pain may be limited (e.g. results might not be generalisable to diverse or male identifying individuals or people from varied cultural backgrounds). To address this important limitation, it will be important for future research to explore whether rates and functions of PICE differ, for example, amongst gender and ethnicity groups, and to gain an understanding of who may be particularly vulnerable to engaging in comfort eating. Despite this relevant limitation, the current results provide valuable insight into eating behaviours in chronic pain, particularly considering that women are more likely to report chronic pain (Rustøen et al., 2004) and endorse higher rates of comfort eating (Adam & Epel, 2007).

While our study has captured some interesting information regarding the self-reported functions of PICE, we were limited by the inflexibility of survey-based data collection and may not have captured the full-spectrum of functions of comfort eating in chronic pain. Future research may benefit from employing qualitative approaches to gain important insights about people’s experiences with PICE and emotional eating including gathering qualitative data on the reasons why people eat (or do not eat) in response to a pain flare-up. A further limitation of the survey-based design is that it might not have captured the true functions of PICE due to recall bias. It is recommended that future studies employ methods such as ecological momentary analysis to collect contemporaneous data on the frequency and function of PICE.

Additionally, our study has been limited by the online nature of the data collection. While this method allowed for easy and wide distribution of our survey flyer to relevant population groups, online studies can be more vulnerable to participant misrepresentation (especially when there is a monetary incentive for participation). It will be important for future research to verify the results of this study using alternative recruitment approaches such as collecting data from participants attending real-world clinical settings such as pain treatment centres. Another interesting avenue for future research to explore is the similarities and differences between PICE, emotional eating and binge eating within chronic pain sufferers to determine how much these constructs overlap.

## Conclusions

In conclusion, this study has further confirmed that comfort eating is both a common and frequently used coping strategy for those with chronic pain. This study sought to better understand why individuals engage in comfort eating finding in response to pain flare-ups and our results indicated that comfort eating may serve to provide a pleasurable experience as well as to distract from the pain flare-up and reduce emotions. Considering that the prevalence of obesity, chronic pain and binge eating are all expected to rise (da Luz et al., 2017; Pain Australia, 2019; Sarma et al., 2021), these findings shed further light on PICE and the function that it serves for individuals with chronic pain.

## Data Availability

The datasets used and/or analysed during the current study are available from the corresponding author on request.
